# Neoadjuvant nivolumab + T-VEC combination therapy for resectable early stage or metastatic (IIIB-IVM1a) melanoma with injectable disease: study protocol of the NIVEC trial

**DOI:** 10.1186/s12885-022-09896-4

**Published:** 2022-08-04

**Authors:** Maartje W. Rohaan, Emma H. A. Stahlie, Viola Franke, Lisanne P. Zijlker, Sofie Wilgenhof, Vincent van der Noort, Alexander C. J. van Akkooi, John B. A. G. Haanen

**Affiliations:** 1 Department of Medical Oncology, Netherlands Cancer Institute, Plesmanlaan 121, 1066CX Amsterdam, The Netherlands; 2 Department of Surgical Oncology, Netherlands Cancer Institute, Plesmanlaan 121, 1066CX Amsterdam, The Netherlands; 3 Department of Biometrics, Netherlands Cancer Institute, Plesmanlaan 121, 1066CX Amsterdam, The Netherlands

**Keywords:** Melanoma, Early metastatic, Neoadjuvant, Talimogene laherparepvec, Nivolumab, Immune checkpoint inhibitor, High-risk, Resectable

## Abstract

**Background:**

Trials investigating neoadjuvant treatment with immune checkpoint inhibitors (ICI) in patients with melanoma have shown high clinical and pathologic response rates. Treatment with talimogene laherparepvec (T-VEC), a modified herpes simplex virus type-1 (HSV-1), is approved for patients with unresectable stage IIIB-IVM1a melanoma and has the potential to make tumors more susceptible for ICI. Combination ICI and intralesional T-VEC has already been investigated in patients with unresectable stage IIIB-IV disease, however, no data is available yet on the potential benefit of this combination therapy in neoadjuvant setting.

**Methods:**

This single center, single arm, phase II study aims to show an improved major pathologic complete response (pCR) rate, either pCR or near-pCR, up to 45% in 24 patients with resectable stage IIIB-IVM1a melanoma upon neoadjuvant combination treatment with intralesional T-VEC and systemic nivolumab (anti-PD-1 antibody). Patients will receive four courses of T-VEC up to 4 mL (first dose as seroconversion dose) and three doses of nivolumab (240 mg flatdose) every 2 weeks, followed by surgical resection in week nine. The primary endpoint of this trial is pathologic response rate. Secondary endpoints are safety, the rate of delay of surgery and event-free survival. Additionally, prognostic and predictive biomarker research and health-related quality of life evaluation will be performed.

**Discussion:**

Intralesional T-VEC has the capacity to heighten the immune response and to elicit an abscopal effect in melanoma in combination with ICI. However, the potential clinical benefit of T-VEC plus ICI in the neoadjuvant setting remains unknown. This is the first trial investigating the efficacy and safety of neoadjuvant treatment of T-VEC and nivolumab followed by surgical resection in patients with stage IIIB-IVM1a melanoma, with the potential of high pathologic response rates and acceptable toxicity.

**Trial registration:**

This trial was registered in the European Union Drug Regulating Authorities Clinical Trials Database (EudraCT- number: 2019–001911-22) and the Central Committee on Research Involving Human Subjects (NL71866.000.19) on 4th June 2020.

Secondary identifying number: NCT04330430.

## Background

The worldwide incidence of melanoma is still increasing and accounted for more than 57.000 deaths in 2020 [1]. The prognosis of patients is clearly correlated with disease stage, but since the introduction of the current standard systemic treatments comprising targeted therapies and immune checkpoint inhibitors (ICI), 5-year overall survival (OS) rates up to 50% have been reached in patients with unresectable stage IIIC-IV melanoma in clinical trials [2, 3]. For patients with high-risk stage IIIB, IIIC, IIID (AJCC 8th edition) melanoma, the prognosis is slightly better, with 5-year OS rates of 83, 69 and 32% respectively, although it must be noted that this is prior to effective adjuvant therapy becoming available [4]. Adjuvant targeted therapy or ICI has shown major improvements in relapse-free survival (RFS) in patients with resectable stage III or IV melanoma [5–8] and is currently standard of care.

Next to the currently available systemic therapies for patients with advanced melanoma, local oncolytic viral immunotherapy with talimogene laherparepvec (T-VEC) has been approved for the treatment of patients with unresectable stage IIIB-IVM1a melanoma with cutaneous, subcutaneous or nodal metastases [9]. T-VEC, a modified herpes simplex virus type-1 (HSV-1), is administered intralesionally and resulted in high and durable response rates with a mild toxicity profile, consisting mostly of fatigue and influenza-like symptoms, confirmed in real-world setting [10–12]. However, there is still a group of patients that has no (durable) benefit upon these currently approved systemic and local treatment options.

A possible way to overcome this resistance to therapy is combination of treatments. Although single agent T-VEC has already proven its efficacy, evidence is also emerging that combination of T-VEC with ICI could have an additive anti-tumor effect. This is demonstrated by two clinical trials treating patients with unresectable stage IIIB-IV melanoma with T-VEC in combination with either ipilimumab, an anti-CTLA-4 antibody, or pembrolizumab, an anti-PD-1 antibody, reaching objective response rates of 39% [13] and 62% [14] respectively. In both trials, the responses were not limited to the injected lesions, suggesting an abscopal effect of T-VEC in combination with ICI. In the phase Ib trial by Ribas et al. [14], 21 patients with unresectable stage IIIB-IV cutaneous melanoma were treated with intralesional T-VEC and systemic pembrolizumab. The majority of patients showed increases in circulating CD4^+^ and CD8^+^ T cells after T-VEC alone, with no significant further increase after pembrolizumab administration. Additionally, increases in CD8^+^ T cells, IFN-γ gene and PD-L1 protein expression were seen within the tumors of responding patients treated with T-VEC and pembrolizumab compared to baseline. These changes in the tumor microenvironment have also been observed by multiple other preclinical and clinical trials [15–17], indicating that T-VEC is able to heighten the immune response and turn an immune desolate “cold” tumor into an immunogenic “hot” tumor, making the tumor more susceptible to ICI treatment. Although the clinical benefit of the combination of T-VEC and pembrolizumab compared to single agent pembrolizumab in patients with unresectable stage IIIB-IVc was not confirmed in the recent phase III trial by Ribas et al. (2021), progression-free survival did seem to be improved in the combination treatment arm and patients with few sites of disease tended to have a better response upon treatment [18], still suggesting a heightened immune response in this patient population. In the previous trials no dose-limiting or novel adverse events (AE) were observed, indicating that combination treatment with T-VEC and ICI can be administered safely to possibly overcome tumor resistance.

Aside from the type of treatment, there is increasing evidence that the timing of the given therapies also plays a key role in the chances of a tumor response [19]. Neoadjuvant therapy, administered prior to surgery, could result in a broader tumor-specific T cell response compared to adjuvant treatment, downsizing of the tumor leading to less extensive surgery, and usage of the pathologic response for fitted (adjuvant) therapy [19, 20]. The benefit of neoadjuvant ICI has been investigated in multiple clinical trials, resulting in high pathologic complete response (pCR) rates of 25–80% [21–24]. Recently, the role of neoadjuvant T-VEC in resectable stage IIIB-IVM1a melanoma has been investigated by Dummer et al. [25] in a randomized study of single agent T-VEC followed by surgery, compared to surgery alone. This trial showed a pCR rate of 17.1% and an improved 2-year RFS and OS for the T-VEC + surgery arm (29.5% vs 16.5 and 88.9% vs 77.4% respectively).

Based on the recent data of neoadjuvant single agent nivolumab and pembrolizumab shown by Amaria et al. [23] and Huang et al. [24], with pCR rates of 25 and 20% respectively, neoadjuvant single agent T-VEC shown by Dummer et al. [25] and the abscopal effect of T-VEC in combination with ICI, we hypothesized that neoadjuvant T-VEC in combination with nivolumab can improve these pCR or pathologic near complete response (near-pCR) rates further. For this, we designed a single arm phase II trial treating patients with stage IIIB-IVM1a melanoma with resectable (sub)cutaneous satellite or in-transit metastases and/or tumor positive lymph nodes with neoadjuvant nivolumab and T-VEC (NIVEC trial). The aim of this trial is to show an improvement in pCR (or near-pCR) rate of 25% compared to the pCR rate observed by Amaria et al. [23] and Huang et al. [24], reaching a pCR rate of 45% in this patient population. In total, 24 patients will be included and evaluated for efficacy and safety. This is the first trial evaluating neoadjuvant T-VEC and ICI combination therapy and may benefit a large group of future melanoma patients.

## Methods

### Study design

The NIVEC trial is an investigator initiated, single-center, single-arm, open-label, phase II study investigating the efficacy and safety of neoadjuvant combination of T-VEC plus nivolumab in patients with resectable stage IIIB-IVM1a melanoma. Patients will be enrolled and treated at the departments of Medical and Surgical Oncology at the Netherlands Cancer Institute (NKI), Amsterdam, The Netherlands, after signing a written informed consent form. A Data and Safety Monitoring Board (DSMB) has been established to monitor the safety of the patients throughout the study.

### Objectives

#### Primary objective

The primary objective of this trial is to investigate the major pathologic complete response rate, either pCR or near-pCR, of neoadjuvant combination of T-VEC and nivolumab, in patients with resectable stage IIIB/C/D/IVM1a melanoma, with the aim to achieve a major pathologic complete response rate of 45%.

#### Secondary objectives

- To investigate the rate of delays or failures (delays ≥14 days) to perform surgery;
- To investigate the effect of neoadjuvant combination of T-VEC and nivolumab on event-free survival (EFS);
- To determine the safety of neoadjuvant combination of T-VEC and nivolumab;
- To acquire tumor material for prognostic and predictive biomarker research, for example CD8, IFN-γ and PD-L1, as well as exploration if expansion of tumor-resident T cells can be observed within the tumor after neoadjuvant treatment.

#### Exploratory objectives

- To assess radiologic response rates according to RECIST 1.1 after neoadjuvant T-VEC and nivolumab at week eight;
- To evaluate health-related quality of life.

### Participants

Patients of ≥18 years with treatment naïve resectable stage IIIB/C/D/IV M1a melanoma according to the American Joint Committee on Cancer (AJCC) 8th edition, a good clinical performance score of 0–1 and < 2 times elevated lactate dehydrogenase levels will be eligible for this trial. Patients must have measurable disease according to RECIST 1.1 [26] and must be a candidate for intralesional therapy with at least one injectable cutaneous, subcutaneous or nodal melanoma lesion (≥ 10 mm in longest diameter) or with multiple injectable lesions that in aggregate have a longest diameter of ≥10 mm. Patients with visceral or brain metastases, prior systemic cancer therapies or a history of other malignancies or autoimmune/infectious diseases will not be eligible. For a full overview of all in- and exclusion criteria, see Table 1.

**Table 1:** In- and exclusion criteria

Inclusion Criteria	Exclusion Criteria
- Adults ≥18 years of age - WHO performance score of 0 or 1 - Cytologically or histologically confirmed diagnosis of stage IIIB/C/D/IVM1a (AJCC 8th edition) melanoma, eligible for surgical resection - Measurable disease according to RECIST 1.1 and must be a candidate for intralesional therapy with at least one injectable cutaneous, subcutaneous or nodal melanoma lesion (≥ 10 mm in longest diameter) or with multiple injectable lesions that in aggregate have a longest diameter of ≥10 mm - Prior isolated limb perfusion is allowed (≥ 12 weeks prior to enrollment) - **Adequate organ function** - LDH < 2 x ULN - INR or PT ≤1.5 x ULN, unless the subject is receiving anticoagulant therapy - WOCBP must use highly effective method(s) of contraception during T-VEC and nivolumab treatment and for a period of 5 months after the last dose of nivolumab - WOCBP must have a negative serum or urine pregnancy test within 72 hours prior to enrollment and within 24 hours prior to the start of nivolumab - Men receiving nivolumab and who are sexually active with WOCBP should use contraception during treatment and for a period of 7 months after the last dose of nivolumab - Men who are sexually active with WOCBP must use any contraceptive method with a failure rate of less than 1% per year - Women who are not of childbearing potential (i.e., who are postmenopausal), or surgically sterile as well as azoospermic men do not require contraception - Patient is capable of understanding and complying with the protocol requirements and has signed the Informed Consent document - Inhaled or topical steroids, and adrenal replacement steroid < 10 mg daily prednisone equivalent, are permitted in the absence of active autoimmune disease	- Liver, bone, lung, brain or other visceral metastases - Prior radiotherapy for melanoma - Prior systemic cancer therapies, including, but not limited to anti-CTLA-4, anti-PD-1, anti-PD-L1 - Other malignancies, except adequately treated and a cancer-related life-expectancy of more than 5 years - Positivity for hepatitis B virus surface antigen or hepatitis C virus ribonucleic acid, indicating acute or chronic infection - Known history of testing positive for HIV or known AIDS - History or evidence of active autoimmune disease that requires high dose systemic treatment. Replacement therapy is not considered a form of systemic treatment - Evidence of clinically significant immunosuppression such as a primary immunodeficiency, a concurrent opportunistic infection, receiving systemic immunosuppressive therapy (> 2 weeks) including oral steroid doses > 10 mg/day of prednisone or equivalent within 7 days prior to enrollment - Active herpetic skin lesions or prior complications of HSV-1 infection - Requirement of intermittent or chronic systemic (intravenous or oral) treatment with an antiherpetic drug, other than intermittent topical use - Previous treatment with T-VEC or any other oncolytic virus - Received live vaccine within 30 days prior to enrollment - Subject has known sensitivity to T-VEC or nivolumab or any of its components to be administered during dosing - Sexually active subjects and their partners unwilling to use male or female latex condom to avoid potential viral transmission during sexual contact while on treatment and within 30 days after treatment with T-VEC - Subjects who are unwilling to minimize exposure with his/her blood or other body fluids to individuals who are at higher risks for HSV-1 induced complications during T-VEC treatment and through 30 days after the last dose of T-VEC - No allergies and adverse drug reaction - History of allergy to study drug components or severe hypersensitivity reaction to any monoclonal antibody - No underlying medical conditions that, in the Investigator’s opinion, will make the administration of study drug hazardous or obscure the interpretation of toxicity determination or adverse events - Use of other investigational drugs before study drug administration 30 days and 5 half-times before study inclusion

*AIDS* acquired immunodeficiency syndrome; *AJCC* American Joint Committee on Cancer; ALT alanine aminotransferase; *AST* aspartate aminotransferase; *CTLA-4* Cytotoxic T-lymphocyte-associated protein-4; *HCG* human chorionic gonadotropin; *HIV* human immunodeficiency virus; *HSV-1* herpes simplex virus type − 1; *INR* international normalization ratio; *LDH* lactate dehydrogenase; *PD-1* programmed cell death protein-1; *PT* prothrombin time; *RECIST* Response Evaluation Criteria in Solid Tumors; *T-VEC* Talimogene laherparepvec; *ULN* upper limit of normal; *WHO* World Health Organization; *WOCBP* women of childbearing potential

### Study procedures and intervention

Prior to enrollment, all patients will be adequately informed by the investigator(s) about participation in the trial. Participation is voluntary and patients are allowed to withdraw from the trial at any time. Following signing of the informed consent form for screening and enrollment into the study, the remainder of the screening procedures and tests will be completed and evaluated before start of treatment. If the screening shows that patients do not fulfill the eligibility criteria anymore, patients will be withdrawn from the study and will be replaced by another patient. See Table 2 for the full schedule of screening assessments and study procedures.

**Table 2:**
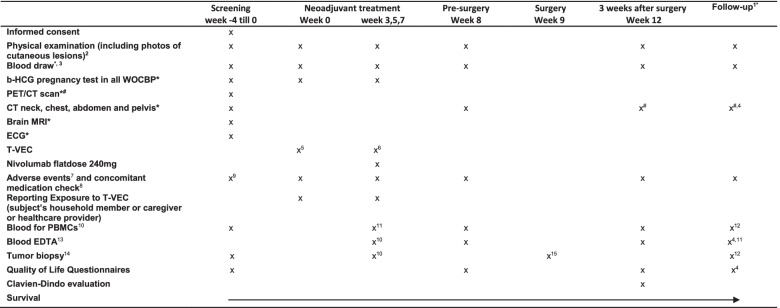
Schedule of enrollment, interventions and assessments

*And when clinically indicated. ^#^According to national/institute’s standards. ^1^Follow-up assessments according to local protocol/standard of care. Every 12 weeks (unless stated other) for up to two years after adjuvant treatment, year 3, 4, 5 according to institute’s standard. ^2^Clinical photographs will be taken of all cutaneous lesions, noting in detail the exact size and location of any (skin) lesions that exist. ^3^Hematology and chemistry. At screening, PT/INR, aPTT and serology (HIV, HbsAG, anti-HCV, HSV, anti-CMV) are also determined. ^4^Every 12 weeks during follow-up. ^5^T-VEC seroconversion dose 10^6^ PFU/mL. ^6^T-VEC dose 10^8^ PFU/mL. ^7^AEs will be graded according to CTCAE v5.0 from moment of signing informed consent. New occurring AEs and any SAEs will reported up to 100 days post last treatment. ^8^Only registration of medication that is used for treatment of immune-related AEs from moment of signing informed consent. ^9^Toxicity from prior treatment will be evaluated. ^10^100 ml heparinized blood (for PBMC). ^11^Only at week 3 prior to first nivolumab. ^12^At relapse. ^13^20 ml EDTA blood (for isolation of plasma for ctDNA). ^14^Three biopsies taken with a 14G needle of thru-cut and will be stored as fresh frozen and formalin-fixed paraffin-embedded material for translational research purposes. At baseline pathological confirmation of melanoma will be performed. ^15^If possible, surgical material should be preserved as fresh frozen and formalin-fixed paraffin-embedded material

*AE* adverse event; *aPTT* activated partial thromboplastin time; *CMV* Cytomegalovirus; *CT* computed tomography; *CTCAE* Common Terminology Criteria for Adverse Events; *ECG* electrocardiogram; *EDTA* Ethylenediaminetetraacetic acid; *HbsAG* hepatitis B virus surface antigen; *HCG* human chorionic gonadotropin; *HCV* hepatitis C virus; *HIV* human immunodeficiency virus; *HSV* herpes simplex virus; *INR* International normalization ratio; *MRI* magnetic resonance imaging; *PBMC* peripheral blood mononuclear cells; *PET* Positron Emission Tomography; *PFU* plaque-forming units; *PTT* partial thromboplastin time; *SAE* Serious Adverse Event; *WOCBP* women of childbearing potential

The total neoadjuvant treatment duration comprises 7 weeks and is followed by surgery in week nine. The treatment schedule is based on four courses of T-VEC and three courses of nivolumab:

- T-VEC: Up to 4 ml T-VEC (first dose 10^6^ plaque-forming units (PFU) per mL as a seroconversion dose, subsequent doses at 10^8^ PFU per mL) in week 0, 3, 5 and 7. The volume of T-VEC to be injected into the tumor depends on the size of the tumor(s) and will be determined based on the longest diameter of each lesion according to manufacturers’ protocol [27];
- Nivolumab: Flatdose of 240 mg nivolumab intravenously every 2 weeks after the first intralesional T-VEC injections (starting at the second T-VEC dose at week 3, followed by the next doses at week 5 and 7).

**Fig. 1:**
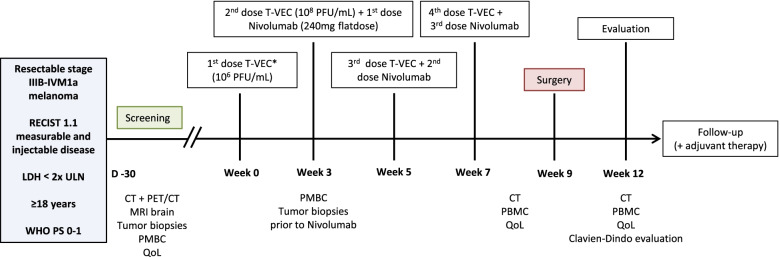
Study Schedule. Blood draw for PBMCs will be performed at screening, week 3, week 9 (prior to surgery), and first evaluation (week 12) and at moment of relapse. Tumor biopsies will be taken at screening, prior to first nivolumab administration and at moment of relapse in all patients for translational research purposes. Biopsies will be preserved and stored as fresh frozen and formalin-fixed paraffin-embedded material at all indicated time points. *The first dose of T-VEC is a seroconversion dose. CT, computed tomography; LDH, lactate dehydrogenase; MRI, magnetic resonance imaging; PBMC, peripheral blood mononuclear cells; PET, Positron Emission Tomography; PFU, plaque-forming units; QoL, quality of life questionnaires; RECIST, Response evaluation criteria in solid tumors; T-VEC, Talimogene laherparepvec; ULN, upper limit of normal; WHO PS, World Health Organization performance status

T-VEC will be administered first, in order to achieve the best synergistic effect with influx of CD8^+^ T cells [14]. Patients will be offered adjuvant treatment as standard of care following surgery. See Fig. 1 for a full overview of the course of the trial.

### Evaluation and follow-up of participants

Prior to each treatment course, physical examination, laboratory analyses and evaluation of toxicity will be performed in each patient. Additionally, photos of cutaneous lesions will be taken. At baseline, before first nivolumab administration, time of surgery and moment of potential relapse, blood- and tumor samples will be collected for translational research purposes. Health-related quality of life assessment will be performed at baseline, prior to surgery, at week 12 and every follow-up.

Prior to surgery, patients will undergo physical examination, laboratory analyses and a Computed Tomography (CT) scan. Following surgery, patients will be evaluated every 12 weeks during the first 2 years after the end of adjuvant treatment by physical examination, laboratory analyses, CT or PET-CT (according to center's discretion). Subsequent structured follow-up will be according to the current national melanoma guidelines. In case of progression to unresectable stage III or stage IV melanoma, follow-up data will be collected until first systemic treatment dose. After initiation of systemic treatment, only the OS data will be collected. See Fig. 1 and Table 2 for all specific time points and study assessments.

### Study endpoints

#### Primary endpoint

The primary endpoint of this trial is the pathologic response rate according to central revision by pathology of NKI, determined after resection following neoadjuvant T-VEC and nivolumab treatment.

#### Secondary endpoints

- Rate of delay of surgery > 14 days and rate of failure to perform surgery defined as no surgery at all;
- EFS, defined as time from randomization to any of the following events: progression of disease that precludes surgery, local or distant recurrence, or death due to any cause;
- Safety of neoadjuvant combination of T-VEC and nivolumab;
- Description of possible prognostic and predictive biomarkers, for example CD8, IFN-γ and PD-L1, and exploration of expansion of tumor-resident T cells.

#### Exploratory endpoints

- Response rate according to RECIST 1.1 at week eight;
- Quality of life as measured by EORTC QLQ-C30 and the Melanoma Surgery Subscale of the FACT-M.

### Evaluation of efficacy

All patients included in the trial who received at least one cycle of therapy will be assessed for pathologic and radiologic response to treatment. Each patient will be assigned one of the following categories for pathologic response based on the resection specimen at week nine: pCR, near-pCR (< 10% vital tumor remaining), pathologic partial response (pPR; < 50% vital tumor remaining), pathologic non-response (pNR) or unknown (not assessable, insufficient data), according to guidelines of the International Neoadjuvant Melanoma Consortium (INMC) [28].

Furthermore, objective radiologic tumor response according to RECIST 1.1 will be measured with CT scans at baseline, week eight and every follow-up. Patients’ response will be classified as “unknown” if insufficient data were collected to allow evaluation per these criteria.

### Safety evaluation

All patients that have received at least one cycle of T-VEC + nivolumab will be evaluated for toxicity according to the National Cancer Institute’s Common Terminology Criteria for Adverse Events version 5.0 (NCI-CTCAE v.5.0) from the moment of signing informed consent until 100 days after last study treatment. Both AEs related and unrelated to treatment will be followed. Additionally, surgical complications will be scored according to the Clavien-Dindo classification [29]. Serious adverse events (SAEs) will be reported to the sponsor and the collaborating party Amgen Inc. (providing T-VEC) within 24 hours. Suspected unexpected serious adverse reactions (SUSARs) will be reported to the Central Committee on Research Involving Human Subjects (CCMO). In addition to the expedited reporting of SUSARs, the sponsor will submit an annual safety report to the CCMO and competent authority. This safety report will also be provided to the subsidising party Amgen Inc. at the time of reporting to the CCMO.

A DSMB has been instituted to monitor the safety of the patients throughout the study. Interim analyses will include only safety information and will be reviewed by the DSMB. After inclusion and treatment of every six patients a report will be provided to the DSMB with an overview of all toxicity observed up until that moment. It is intended not to include any data on response and thus not to look at the primary endpoint before the end of the study.

### Evaluation of health-related quality of life

Health-related quality of life will be assessed with the European Organization for Research and Treatment of Cancer Quality of Life Questionnaire Core 30 (EORTC QLQ-C30), a self-reported questionnaire specifically developed for patients with cancer who are receiving cancer treatment and is the most common quality of life instrument used in melanoma studies [30]. The guideline of Cocks et al. [31] will be used for the interpretation of changes in QLQ-C30 scores. Additionally, the Melanoma Surgery Subscale of the FACT-M [32] will be evaluated in all patients every 12 weeks during the first year and at year two and three.

### Translational research

Tumor material (at baseline, during treatment and the resection specimen), peripheral blood mononuclear cells (PBMCs) and serum will be collected in all patients (see Fig. 1 and Table 2 for all time points) to perform in depth translational research into the mechanism of action of T-VEC in combination with nivolumab, both on the tumor and the tumor micro-environment. The planned analyses of the biopsies and PBMCs will primarily provide the information to underline our hypothesis about the synergistic effect of T-VEC and nivolumab.

### Data collection, management and monitoring

All source data from the trial will be collected and managed in the electronic case report form (eCRF) developed by the datacenter of the NKI via ALEA (FormsVision BV, Abcoude, The Netherlands), a web-based software system. The completed eCRFs must be reviewed and signed by the principal investigator or sub-investigator. The handling of personal data complies with the Dutch Personal Data Protection Act and all data management activities will follow local standard operating procedures. Statistical analysis will be performed after a database lock, after all study data have been collected and cleaned.

According to the Dutch Federation of University Medical Centers, this trial was classified as a ‘low risk’ study. This implies at least one on-site source data verification of the eCRFs and check of the Investigator Study File documents by the clinical research monitor.

### Statistical considerations

We suspect that neoadjuvant combination treatment of T-VEC with nivolumab will achieve a higher major pathologic complete response rate in patients with resectable stage IIIB/C/D-IVM1a melanoma compared to neoadjuvant treatment with either single agent T-VEC or nivolumab. Additionally, we expect that neoadjuvant T-VEC plus nivolumab will result in a higher response rate in our patient population compared to patients with unresectable disease, as the tumor burden is lower. The combination treatment will be considered successful if the major pathologic complete response rate lies significantly above the response rate of 20% obtained with neoadjuvant single agent ICI. For the current neoadjuvant combination study we thus expect a pCR (or near-pCR) rate of 45% for T-VEC combined with nivolumab. An exact binomial test with a nominal 10% two-sided significance level will have 82.7% power to detect the difference between the Null hypothesis proportion, π_0_, of 0.2 and the Alternative proportion, π_1_, of 0.45 when the sample size is 24.

Assuming a surgical success rate of 95%, defined as a complete dissection of the involved lymph nodes and/or all cutaneous/subcutaneous satellite/in-transit disease as planned, including 24 patients will have 88% power to exclude a lower level of 75% with 95% confidence (one-sided). The combination needs to be reconsidered when surgery as planned can no longer be performed due to progressive disease or AEs in more than two patients. Considering the chosen scenario of 24 patients, the study is also powered to analyze this secondary endpoint.

## Discussion

This manuscript describes the clinical trial protocol of the NIVEC trial, the first single-center, single-arm, phase II trial investigating the efficacy and safety of neoadjuvant nivolumab in combination with T-VEC in patients with resectable stage IIIB-IVM1a melanoma. To date, T-VEC in combination with ICI has only been evaluated in clinical trials with patients with unresectable stage IIIB-IVM1c melanoma [13, 14, 18]. It is known that T-VEC is able to modify the tumor microenvironment to make it more susceptible to concurrent ICI [14] and that neoadjuvant treatment has the ability to elicit a broader tumor-specific T cell response [19]. The design of the trial was based on the successful results of earlier neoadjuvant trials with single agent ICI and T-VEC [23–25] and is the first trial to administer this treatment combination in patients with resectable stage IIIB-IVM1a disease, creating a window of potential for improved response rates in this patient population.

In prior neoadjuvant trials in patients with resectable stage III melanoma, such as the OpACIN-neo trial [22], neoadjuvant treatment with three different dosing schedules of ICI with ipilimumab and nivolumab were administered during a period of 6 weeks prior to surgery. In the current trial, a similar neoadjuvant treatment period is followed, however, as the first T-VEC dose concerns a seroconversion dose and the next (first optimal) T-VEC dose can be administered 3 weeks thereafter, the surgery will take place 9 weeks after initiation of the neoadjuvant treatment. As T-VEC is administered every 2 weeks, we suggested the same dosing schedule for T-VEC and nivolumab every 2 weeks for the purpose of this trial.

This dosing schedule will result in a short delay for patients compared to standard treatment, as regular times to surgery in The Netherlands are 4–6 weeks. However, standard of care for this patient population would be surgery followed by adjuvant ICI. In this trial, patients will already have received three courses of ICI in combination with T-VEC prior to surgery. Considering the very high chance of relapse in this patient category (approximately 70–80% risk) [33], any neoadjuvant approach might reduce this risk of relapse, adding to the chance of a more durable response for these patients, comparable to the observed results from the earlier neoadjuvant OpACIN and OpACIN-neo studies [21, 22]. Results from Dummer et al. [25], already demonstrated 25% less chance of recurrence in patients with resectable stage IIIB-IVM1a melanoma when treated with neoadjuvant T-VEC compared to surgery alone. For patients with subcutaneous or in-transit metastases, who are often excluded from ICI neoadjuvant trials [22] and who would otherwise receive locoregional management (followed by adjuvant therapy in case of surgery) or systemic therapy as standard of care, this neoadjuvant approach could be extra beneficial. Additionally, some patients may have the chance to undergo less extensive surgery, or no surgery at all, due to a good clinical response upon neoadjuvant treatment.

Earlier neoadjuvant trials with combination ICI have shown improved pCR rates compared to monotherapy ICI in neoadjuvant setting. However, this was often also associated with higher toxicity profiles [21–24]. Ways to improve neoadjuvant treatment by diminishing toxicity rates whilst maintaining these improved response rates are currently under investigation in multiple clinical trials, evaluating the safety and efficacy of different neoadjuvant combination treatments. In an ongoing phase II trial, the combination of neoadjuvant pembrolizumab and lenvatinib, a multiple kinase inhibitor, is being investigated in patients with resectable stage III/IV melanoma [34] and improved toxicity rates have recently already been reported with neoadjuvant relatlimab, an anti-LAG-3 antibody, in combination with nivolumab in patients with unresectable or metastatic melanoma [35]. Both neoadjuvant single agent nivolumab and T-VEC have shown mild toxicity rates, with grade 3–4 AEs occurring in 8% [23] and 5.5% [25] of patients respectively. We expect the toxicity profile of the combination of T-VEC and nivolumab to be acceptable, as T-VEC combined with pembrolizumab already showed no increase in toxicity rate compared to single agent ICI in patients with unresectable stage IIIB-IV melanoma [14, 18].

In conclusion, this is the first trial to investigate neoadjuvant T-VEC and nivolumab in patients with resectable stage IIIB-IVM1a melanoma. It has the potential to show improved efficacy with acceptable toxicity profiles, compared to single agent or ICI combination neoadjuvant treatment, and could thus lead to a potential novel viable treatment option for this patient population.

### Current trial status

The NIVEC trial was open for accrual on 7th July 2020 and a total of 13 of the planned 24 patients were enrolled in the trial on 31st May 2022.

## Data Availability

Not applicable.

## Abbreviations

AE: Adverse event
AJCC: American Joint Committee on Cancer
CCMO: Central Committee on Research Involving Human Subjects
CTCAE: Common Terminology Criteria for Adverse Events
CTLA-4: Cytotoxic T-lymphocyte-associated protein-4
DSMB: Data and Safety Monitoring Board
eCRF: Electronic case report form
EFS: Event-free survival
EORTC QLQ-C30: European Organization for Research and Treatment of Cancer Quality of Life Questionnaire Core 30
EudraCT: European Union Drug Regulating Authorities Clinical Trials Database
FACT-M: Functional Assessment of Cancer Therapy – Melanoma
GCP: Good Clinical Practice
HSV-1: Herpes simplex virus type-1
ICI: Immune checkpoint inhibition
IFN: Interferon
INMC: International Neoadjuvant Melanoma Consortium
near-pCR: Pathologic near complete response
NKI: Netherlands Cancer Institute
OS: Overall survival
PBMC: Peripheral blood mononuclear cells
pCR: Pathologic complete response
PD: Progressive disease
PD-1: Programmed cell death protein-1
PD-L1: Programmed cell death ligand-1
PFU: Plaque-forming units
pNR: Pathologic non-response
pPR: Pathologic partial response
RECIST: Response evaluation criteria in solid tumors
RFS: Relapse-free survival
SAE: Serious Adverse Event
SUSAR: Suspected unexpected serious adverse reaction
T-VEC: Talimogene laherparepvec
WMO: Medical Research Involving Human Subjects Act

## References

[1] Sung H, Ferlay J, Siegel RL, Laversanne M, Soerjomataram I, Jemal A (2021). Global Cancer statistics 2020: GLOBOCAN estimates of incidence and mortality worldwide for 36 cancers in 185 countries. CA Cancer J Clin.

[2] Robert C, Grob JJ, Stroyakovskiy D, Karaszewska B, Hauschild A, Levchenko E (2019). Five-year outcomes with Dabrafenib plus Trametinib in metastatic melanoma. N Engl J Med.

[3] Larkin J, Chiarion-Sileni V, Gonzalez R, Grob JJ, Rutkowski P, Lao CD (2019). Five-year survival with combined Nivolumab and Ipilimumab in advanced melanoma. N Engl J Med.

[4] Gershenwald JE, Scolyer RA (2018). Melanoma staging: American joint committee on Cancer (AJCC) 8th edition and beyond. Ann Surg Oncol.

[5] Weber J, Mandala M, Del Vecchio M, Gogas HJ, Arance AM, Cowey CL (2017). Adjuvant Nivolumab versus Ipilimumab in resected stage III or IV melanoma. N Engl J Med.

[6] Eggermont AMM, Blank CU, Mandala M, Long GV, Atkinson V, Dalle S (2018). Adjuvant Pembrolizumab versus placebo in resected stage III melanoma. N Engl J Med.

[7] Long GV, Hauschild A, Santinami M, Atkinson V, Mandalà M, Chiarion-Sileni V (2017). Adjuvant Dabrafenib plus Trametinib in stage III BRAF-mutated melanoma. N Engl J Med.

[8] Ascierto PA, Del Vecchio M, Mandalá M, Gogas H, Arance AM, Dalle S (2020). Adjuvant nivolumab versus ipilimumab in resected stage IIIB-C and stage IV melanoma (CheckMate 238): 4-year results from a multicentre, double-blind, randomised, controlled, phase 3 trial. Lancet Oncol..

[9] Andtbacka RHI, Collichio F, Harrington KJ, Middleton MR, Downey G, Ӧhrling K (2019). Final analyses of OPTiM: a randomized phase III trial of talimogene laherparepvec versus granulocyte-macrophage colony-stimulating factor in unresectable stage III-IV melanoma. J Immunother Cancer.

[10] Franke V, Berger DMS, Klop WMC, van der Hiel B, van de Wiel BA, Ter Meulen S (2019). High response rates for T-VEC in early metastatic melanoma (stage IIIB/C-IVM1a). Int J Cancer.

[11] Stahlie EHA, Franke V, Zuur CL, Klop WMC, van der Hiel B, Van de Wiel BA (2021). T-VEC for stage IIIB-IVM1a melanoma achieves high rates of complete and durable responses and is associated with tumor load: a clinical prediction model. Cancer Immunol Immunother.

[12] van Akkooi ACJ, Haferkamp S, Papa S, Franke V, Pinter A, Weishaupt C (2021). A retrospective chart review study of real-world use of Talimogene Laherparepvec in Unresectable stage IIIB-IVM1a melanoma in four European countries. Adv Ther.

[13] Chesney J, Puzanov I, Collichio F, Singh P, Milhem MM, Glaspy J (2018). Randomized, open-label phase II study evaluating the efficacy and safety of Talimogene Laherparepvec in combination with Ipilimumab versus Ipilimumab alone in patients with advanced. Unresectable MelanomaJ Clin Oncol.

[14] Ribas A, Dummer R, Puzanov I, VanderWalde A, Andtbacka RHI, Michielin O (2017). Oncolytic Virotherapy promotes Intratumoral T cell infiltration and improves anti-PD-1 immunotherapy. Cell..

[15] Moesta AK, Cooke K, Piasecki J, Mitchell P, Rottman JB, Fitzgerald K (2017). Local delivery of OncoVEX (mGM-CSF) generates systemic antitumor immune responses enhanced by cytotoxic T-lymphocyte-associated protein blockade. Clin Cancer Res.

[16] Gogas H, Samoylenko I, Schadendorf D, Gutzmer R, Grob JJ, Sacco JJ (2018). 1246PD - Talimogene laherparepvec (T-VEC) treatment increases intratumoral effector T-cell and natural killer (NK) cell density in noninjected tumors in patients (pts) with stage IIIB–IVM1c melanoma: evidence for systemic effects in a phase II, single-arm study. Ann Oncol.

[17] Kaufman HL, Kim DW, DeRaffele G, Mitcham J, Coffin RS, Kim-Schulze S (2010). Local and distant immunity induced by intralesional vaccination with an oncolytic herpes virus encoding GM-CSF in patients with stage IIIc and IV melanoma. Ann Surg Oncol.

[18] Ribas A, Chesney J, Long GV, Kirkwood JM, Dummer R, Puzanov I (2021). 1037O MASTERKEY-265: a phase III, randomized, placebo (Pbo)-controlled study of talimogene laherparepvec (T) plus pembrolizumab (P) for unresectable stage IIIB–IVM1c melanoma (MEL). Ann Oncol.

[19] Versluis JM, Long GV, Blank CU (2020). Learning from clinical trials of neoadjuvant checkpoint blockade. Nat Med.

[20] Liu J, Blake SJ, Yong MC, Harjunpää H, Ngiow SF, Takeda K (2016). Improved efficacy of Neoadjuvant compared to adjuvant immunotherapy to eradicate metastatic disease. Cancer Discov.

[21] Blank CU, Rozeman EA, Fanchi LF, Sikorska K, van de Wiel B, Kvistborg P (2018). Neoadjuvant versus adjuvant ipilimumab plus nivolumab in macroscopic stage III melanoma. Nat Med.

[22] Rozeman EA, Menzies AM, van Akkooi ACJ, Adhikari C, Bierman C, van de Wiel BA (2019). Identification of the optimal combination dosing schedule of neoadjuvant ipilimumab plus nivolumab in macroscopic stage III melanoma (OpACIN-neo): a multicentre, phase 2, randomised, controlled trial. Lancet Oncol.

[23] Amaria RN, Reddy SM, Tawbi HA, Davies MA, Ross MI, Glitza IC (2018). Neoadjuvant immune checkpoint blockade in high-risk resectable melanoma. Nat Med.

[24] Huang AC, Orlowski RJ, Xu X, Mick R, George SM, Yan PK (2019). A single dose of neoadjuvant PD-1 blockade predicts clinical outcomes in resectable melanoma. Nat Med.

[25] Dummer R, Gyorki DE, Hyngstrom J, Berger AC, Conry R, Demidov L (2021). Neoadjuvant talimogene laherparepvec plus surgery versus surgery alone for resectable stage IIIB-IVM1a melanoma: a randomized, open-label, phase 2 trial. Nat Med.

[26] Eisenhauer EA, Therasse P, Bogaerts J, Schwartz LH, Sargent D, Ford R (2009). New response evaluation criteria in solid tumours: revised RECIST guideline (version 1.1). Eur J Cancer.

[27] Highlights of prescribing information, IMLYGIC (talimogene laherparepvec). Available at: https://www.pi.amgen.com/-/media/Project/Amgen/Repository/pi-amgen-com/imlygic/imlygic_pi.pdf.

[28] Tetzlaff MT, Messina JL, Stein JE, Xu X, Amaria RN, Blank CU (2018). Pathological assessment of resection specimens after neoadjuvant therapy for metastatic melanoma. Ann Oncol.

[29] Dindo D, Demartines N, Clavien PA (2004). Classification of surgical complications: a new proposal with evaluation in a cohort of 6336 patients and results of a survey. Ann Surg.

[30] Aaronson NK, Ahmedzai S, Bergman B, Bullinger M, Cull A, Duez NJ (1993). The European Organization for Research and Treatment of Cancer QLQ-C30: a quality-of-life instrument for use in international clinical trials in oncology. J Natl Cancer Inst.

[31] Cocks K, King MT, Velikova G, Martyn St-James M, Fayers PM, Brown JM (2011). Evidence-based guidelines for determination of sample size and interpretation of the European organisation for the research and treatment of Cancer quality of life questionnaire Core 30. J Clin Oncol.

[32] Askew RL, Xing Y, Palmer JL, Cella D, Moye LA, Cormier JN (2009). Evaluating minimal important differences for the FACT-melanoma quality of life questionnaire. Value Health.

[33] Svedman FC, Pillas D, Taylor A, Kaur M, Linder R, Hansson J (2016). Stage-specific survival and recurrence in patients with cutaneous malignant melanoma in Europe - a systematic review of the literature. Clin Epidemiol.

[34] Gonzalez M, Menzies AM, Pennington T, Saw RP, Spillane AJ, Stretch J (2020). A phase II study of neoadjuvant pembrolizumab and lenvatinib for resectable stage III melanoma: the neopele study. J Clin Oncol.

[35] Amaria RN, Postow MA, Tetzlaff MT, Ross MI, Glitza IC, McQuade JL (2021). Neoadjuvant and adjuvant nivolumab (nivo) with anti-LAG3 antibody relatlimab (rela) for patients (pts) with resectable clinical stage III melanoma. J Clin Oncol.

